# The impact of hormones on emotional and social development: a study in adolescent daughters of women with polycystic ovary syndrome

**DOI:** 10.1192/j.eurpsy.2024.275

**Published:** 2024-08-27

**Authors:** J. P. del Río, F. Dabed, J. Díaz, A. Ladrón de Guevara, P. Gaspar, A. Maturana, N. Crisosto

**Affiliations:** ^1^Department of Child and Adolescent Psychiatry; ^2^Endocrinology and Metabolism Laboratory, Faculty of Medicine; ^3^Translational Psychiatry Laboratory (Psiquislab), University Psychiatric Clinic, Universidad de Chile; ^4^Endocrinology Unit, Department of Medicine, Clínica Alemana de Santiago, Faculty of Medicine, Universidad del Desarrollo, Santiago, Chile

## Abstract

**Introduction:**

Polycystic Ovarian Syndrome (PCOS) is the most prevalent endocrine disorder in adolescents. It affects brain maturation, specially in highly neuronal plasticity periods However, there is a lack of information about the impact of this exposure during brain plasticity windows.

**Objectives:**

Characterize the consequences of hyperandrogenism in emotional status and social cognition (SC) on adolescents daughters of women with PCOS (dPCOS).

**Methods:**

Analytical cross sectional study. dPCOS and controls between ages of 12 to 25 years old were recruited. Participants underwent a complete clinical evaluation, plasmatic hormones determinations (including total testosterone, SHBG, androstenedione and 17-OH-progesterone) and ovarian ultrasound characterization. SC was estimated by: measurements of affects (PANAS), strength and difficulties (SDQ), self-reported empathy (EQ/SQ and AQ), and gaze patterns for autonomic response measurement via Eye-Tracking.

**Results:**

33 participants were recruited, 15 cases and 18 controls. Median age was 17 and 18 years, respectively. The dPCOS presented a larger anogenital distance (cm) (9.7 vs 7.8; p=0.014), Ferryman-Gallwey score mean (13.0 vs 2.0; p=<0.001) and free androgen index value (7.5 vs 4.1; p=0.004), suggesting hyperandrogenism exposure during intrauterine and adolescence periods. Regarding SC, dPCOS exhibited a predominantly negative affective status (PANAS 8.0 vs 2.0, p=0.049) and a higher score in socio-emotional problems (SDQ 2,5 vs 1,5; p=0,047). The eye-tracking registration showed that dPCOS presentes longer time to first fixation in areas of interest (s) (0,35 vs 0,28; p=0,037), which was associated with a worse endpoint in emotional recognition (aR2=-0,920; f=19,48; Pr >|t|=<0,049). Furthermore, the 2D:4D ratio (intrauterine marker of androgen exposure) was correlated with a predominance of negative affect (rho=0,51; p=0,019) and less prosocial behaviors (coef=-2,39; P>|t|=0,049).

**Conclusions:**

Clinical and hormonal markers suggest that dPCOS are exposed to hyperandrogenism during the most critical neuroplasticity periods. This exposure is associated with negative affects, more social-emotional difficulties and less score on emotional recognition and prosocial behavior. Due to a high psychiatric comorbidity in PCOS patients, these findings are relevant and emphasize the importance of early mental health treatment in these patients.

**Disclosure of Interest:**

None Declared

